# Digital Education for Health Professions on Smoking Cessation Management: Systematic Review by the Digital Health Education Collaboration

**DOI:** 10.2196/13000

**Published:** 2019-03-04

**Authors:** Monika Semwal, Penny Whiting, Ram Bajpai, Shweta Bajpai, Bhone Myint Kyaw, Lorainne Tudor Car

**Affiliations:** 1 Centre for Population Health Sciences Lee Kong Chian School of Medicine Nanyang Technological University Singapore Singapore; 2 Bristol Medical School University of Bristol Bristol United Kingdom; 3 National Institute for Health Research Collaboration for Leadership in Applied Health Research and Care West University Hospitals Bristol NHS Foundation Trust Bristol United Kingdom; 4 Family Medicine and Primary Care Lee Kong Chian School of Medicine Nanyang Technological University Singapore Singapore; 5 Department of Primary Care and Public health School of Public health Imperial College London London United Kingdom

**Keywords:** evidence-based practice, health personnel, learning, systematic review, smoking cessation

## Abstract

**Background:**

Tobacco smoking, one of the leading causes of preventable death and disease, is associated with 7 million deaths every year. This is estimated to rise to more than 8 million deaths per year by 2030, with 80% occurring in low- and middle-income countries. Digital education, teaching, and learning using digital technologies have the potential to increase educational opportunities, supplement teaching activities, and decrease distance barriers in health professions education.

**Objective:**

The primary objective of this systematic review was to evaluate the effectiveness of digital education compared with various controls in improving learners’ knowledge, skills, attitudes, and satisfaction to deliver smoking cessation therapy. The secondary objectives were to assess patient-related outcomes, change in health professionals’ practice or behavior, self-efficacy or self-rated competence of health professionals in delivering smoking cessation therapy, and cost-effectiveness of the interventions.

**Methods:**

We searched 7 electronic databases and 2 trial registers for randomized controlled trials published between January 1990 and August 2017. We used gold standard Cochrane methods to select and extract data and appraise eligible studies.

**Results:**

A total of 11 studies (number of participants, n=2684) were included in the review. All studies found that digital education was at least as effective as traditional or usual learning. There was some suggestion that blended education results in similar or greater improvements in knowledge (standardized mean difference, SMD=0.19, 95% CI −0.35 to 0.72), skill (SMD=0.58, 95% CI 0.08-1.08), and satisfaction (SMD=0.62, 95% CI 0.12-1.12) compared with digital education or usual learning alone. There was also some evidence for improved attitude (SMD=0.45, 95% CI 0.18-0.72) following digital education compared with usual learning. Only 1 study reported patient outcomes and the setup cost of blended education but did not compare outcomes among groups. There were insufficient data to investigate what components of the digital education interventions were associated with the greatest improvements in learning outcomes.

**Conclusions:**

The evidence suggests that digital education is at least as effective as usual learning in improving health professionals’ knowledge and skill for delivering smoking cessation therapy. However, limitations in the evidence base mean that these conclusions should be interpreted with some caution.

**Trial Registration:**

PROSPERO CRD42016046815; https://www.crd.york.ac.uk/prospero/display_record.php?RecordID=46815

## Introduction

Tobacco smoking, one of the leading causes of preventable death and disease, is associated with 7 million deaths every year. This is estimated to rise to more than 8 million deaths per year by 2030, with 80% occurring in low- and middle-income countries [1]. There is promising evidence to show that interventions delivered by health professionals are effective in preventing and stopping tobacco smoking [2-4]. Health professionals’ advice has been shown to increase attempts to stop smoking and use medications aimed at stopping smoking. An intervention as brief as 3 min can result in a significant increase in smoking cessation rates [5]. However, the lack of relevant knowledge and skill is a significant barrier reported by health professionals, preventing them from providing smoking cessation advice to their patients who are tobacco smokers [6,7]. Timely and cost-effective training and education are essential to ensure that health professionals have appropriate knowledge and skill to deliver smoking cessation–related interventions.

Digital education, teaching, and learning using digital technologies have the potential to increase educational opportunities, supplement teaching activities, and decrease distance barriers in health professional education [8]. Encompassing a broad spectrum of interventions, digital education can combine self-directed learning with practical skill-based training to successfully deliver smoking cessation therapy to health professionals [9,10]. The different intervention modalities such as mobile phone apps, computer-assisted learning, simulation-based learning, and social networking can create active learning environments that provide real-time feedback and enable health professionals to participate in in-depth discussions of relevant topics. Digital education also offers the opportunity for professionals to become specialists in comprehensive smoking cessation by delivering more smoking cessation training than usually provided in the traditional classroom setting [8,11,12].

Previous systematic reviews that have evaluated digital education for smoking cessation and prevention have focused on interventions aimed at patients rather than training health professionals [13-16]. This systematic review is 1 of a series of reviews evaluating the scope for implementation and potential impact of a wide range of digital health education interventions for pre and postregistration health professionals. The primary objective of this systematic review is to evaluate the effectiveness of digital education compared with various controls in improving learners’ knowledge, skills, attitudes, and satisfaction to deliver smoking cessation therapy. The secondary objectives are to assess patient-related outcomes, change in health professionals’ practice or behavior, self-efficacy or self-rated competence of health professionals in delivering smoking cessation therapy, and cost-effectiveness of the interventions.

If digital education is at least as effective as standard face-to-face learning methods, then there is the potential for digital education to be used to deliver training in smoking cessation therapy with associated benefits such as being able to reach a much larger audience and allowing more flexibility in when and where the training is undertaken. The evidence could be used to make recommendations regarding the optimal digital education approach to train health professionals to deliver smoking cessation therapy.

## Methods

The Cochrane recommendations for the conduct of systematic review were followed, and this review is reported according to the Preferred Reporting Items for Systematic Reviews and Meta-Analyses guidance [17,18]. A protocol detailing the review methods was also produced and followed throughout the review. This protocol was registered with PROSPERO (ID: CRD42016046815). For a detailed description of the methodology, please refer to the study by Car et al [19].

### Data Sources

This review is a part of a global evidence synthesis initiative on digital health professions education for which a wider search strategy was developed (Multimedia Appendix 1). The following databases were searched from January 1990 to August 2017: Medical Literature Analysis and Retrieval System Online (Ovid), Excerpta Medica dataBASE (Ovid), Central Register of Controlled Trials (CENTRAL; Cochrane Library), PsycINFO (Ovid), Education Resources Information Center (Ovid), Cumulative Index to Nursing and Allied Health Literature (EBSCO), and Web of Science Core Collection (Thomson Reuters). The search was limited to studies reported after 1990, as before this, the use of computers was limited to very basic tasks.

No language or publication restrictions were applied. We searched reference lists of all included studies and relevant systematic reviews. The International Clinical Trials Registry Platform Search Portal and Current Controlled Trials metaRegister of Controlled Trials were also searched to identify unpublished or ongoing trials, as well as meeting abstracts and PhD theses. We contacted study authors of included studies to ask if they were aware of other relevant studies and to provide full reports where these were not identified by the searches. Search results from different sources were combined in a single library and duplicate records were removed.

### Study Selection

We included individual or cluster randomized trials (cRCTs) that compared digital education to self and usual or traditional learning or other forms of digital education to train pre or postregistration health professionals to deliver smoking cessation therapy. Health professionals with qualifications listed in the Health Field of Education and Training (091) in the International Standard Classification of Education [20] were included. We excluded studies of students and/or practitioners of traditional, alternative, and complementary medicine. Digital education interventions could be delivered as the main mode of the education intervention or as a part of a complex, multicomponent intervention (ie, blended education). We accepted any type of digital education such as offline and Web-based, computer-based digital education, Serious Gaming and Gamification, massive open online courses, virtual reality environments, virtual patient simulations, psychomotor skill trainers, and mobile learning or mLearning [21-25]. No restrictions on outcomes were applied.

Moreover, 2 reviewers (MS and SB) independently screened titles and abstracts identified by the searches. Full texts of potentially relevant articles were obtained and independently assessed for inclusion by 2 reviewers (MS and SB). Where data were missing or incomplete, authors were contacted for additional information [26,27]. Any disagreements were resolved through discussions between the 2 reviewers with a third reviewer acting as an arbiter (RB).

### Data Extraction and Quality Assessment

A total of 2 reviewers (MS and SB) independently extracted data using a standardized data extraction form, which was piloted and amended on the basis of feedback. Data were extracted on study design, participants’ demographics, type of digital education, intervention content, and outcomes. We contacted study authors of the included studies in case of any unclear or missing information. Disagreements between reviewers were resolved by discussion. A third reviewer (RB) acted as an arbiter in cases where disagreements persisted.

Data on the following primary outcomes were extracted:

1. Learners’ knowledge postintervention. Knowledge is defined as the learners’ factual or conceptual understanding measured using difference in pre and posttest scores.
2. Learners’ skill postintervention. Skill is defined as the learners’ ability to demonstrate a procedure or technique in an educational setting.
3. Learners’ attitudes postintervention toward digital education or toward new clinical knowledge and skill or patients (eg, awareness of moral and ethical responsibilities involved in patient contact). Attitude is defined as the tendency to respond positively or negatively toward the intervention.
4. Learners’ satisfaction postintervention with the learning intervention (eg, retention rates, dropout rates, and survey satisfaction scores). This is defined as the level of approval when comparing the perceived performance of digital education with one’s expectations.

We also extracted data on the following secondary outcomes:

1. Patient-related outcomes (eg, heaviness of smoking index, number of patients who are stopping smoking).
2. Change in health professionals’ practice or behavior.
3. Self-efficacy referred to as self-rated competence of health professionals in delivering smoking cessation therapy.
4. Cost and cost effectiveness of the intervention.

For continuous outcomes, we extracted data to calculate standardized mean difference (SMD) and associated 95% CIs in change from baseline or at follow-up between intervention and control groups. For studies that reported median and range for the various outcomes, we converted this to mean and standard deviation [28]. For dichotomous outcomes, we extracted data to calculate relative risks (RRs) and 95% CIs. Where studies reported more than 1 measure for each outcome, the primary measure as defined by the study authors was extracted.

### Risk of Bias Assessment

The methodological quality of included randomized controlled trials was independently assessed by 2 reviewers (MS and SB) using the Cochrane risk of bias tool, which includes the following domains: random sequence generation, allocation concealment, blinding of participants to the intervention, blinding of outcome assessment, attrition, and selective reporting. We also assessed the additional domain of baseline imbalances [17]. The following additional criteria were included for the assessment of cRCTs: recruitment bias that can occur when individuals are recruited to the trial after the clusters have been randomized, loss of clusters, incorrect analysis, and comparability with individually randomized trials [17].

### Data Synthesis and Analysis

We grouped studies according to outcomes assessed—skill, knowledge, attitude, satisfaction, practice and behavior change, self-efficacy, patient outcomes, and cost. Within these outcomes, we further grouped studies on the basis of intervention (digital education or blended education) and comparison (usual learning or traditional education, blended education, or other forms of digital education)

Heterogeneity was assessed visually using forest plots and by considering differences in participants, interventions, and outcomes across studies. Due to substantial differences among studies, we used a narrative approach to data synthesis. We were unable to identify a clinically meaningful interpretation of effect size in the literature for digital education interventions. Therefore, in line with other research in the field, we present outcomes using postintervention SMD and interpret the effect size using Cohen *rule of thumb* (ie, with 0.2 representing a small effect, 0.5 a moderate effect, and 0.8 a large effect) [17]. For dichotomous outcomes, we summarized RRs and associated 95% CIs across studies. Subgroup analyses were not feasible because of the small number of studies.

## Results

**Figure 1:**
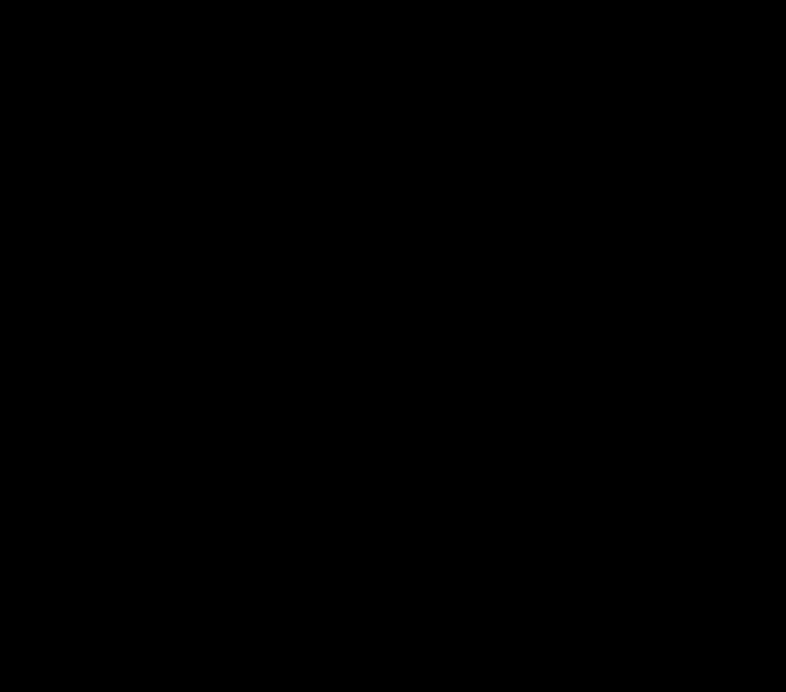
Preferred Reporting Items for Systematic Reviews and Meta-Analyses flow diagram. RCT: randomized controlled trial.

Our search strategy for a series of systematic reviews focusing on different digital health professional education modalities yielded 30,532 unique references. Upon screening of titles and abstracts, we excluded 30,051 ineligible references and retrieved full texts for 22 potentially eligible studies. We excluded 10 studies that did not meet the inclusion criteria: 3 were not randomized trials, 4 did not evaluate digital education intervention, and 3 did not target health professionals. A total of 11 studies (12 reports; 2684 health professionals) were included in the review—8 individually randomized trials and 3 cluster randomized trials. Furthermore, 1 study was reported in 2 separate journal articles [29]. The flow of studies through the systematic review process is shown in Figure 1. Characteristics of the 11 included studies are summarized in Table 1.

All included studies were published in English. In addition, 7 studies focused on postregistration health professionals—3 were restricted to doctors [26,29,34] and 4 included mixed populations of doctors and other health professionals [27,31,35,36]. The remaining 4 studies included preregistration health professionals—2 studies included medical students [33,37] and 2 included pharmacy students [30,32]. Furthermore, 10 studies were conducted in high-income countries, 2 in Australia [26,34], 6 in the United States [29,31-33,35,36], and single studies in the United Kingdom [27] and Switzerland [37]. In addition, 1 study was conducted in Thailand, a middle-income country [30]. A total of 4 studies compared digital education with usual learning, 2 studies compared different digital education interventions [34,35], and 2 studies compared blended education with digital education [36,37]. The 3 cRCTs compared blended education with usual learning [27,29,33].

Several modalities were used to deliver the digital education intervention. Web-based systems were used in 6 studies where participants could access learning materials through a Web gateway [27-31,37]. Of these, 2 studies used computer-based programs, 1 with computerized feedback [34], and the other was an interactive multimedia program [30]. Technology-enabled student response systems or clickers were used to provide instructions in 1 study [32]. Moreover, 5 studies blended digital education components utilizing CD-ROM, computer or Web-based interface with usual learning modalities such as face-to-face interactions, lectures, and seminars [27,29,33,36,37]. Although most interventions focused on improving knowledge about smoking cessation and skill in delivering smoking cessation therapy [26,29,30,33-37], the content of the smoking cessation education varied widely. Table 1 provides a detailed overview of the interventions compared in each study.

**Table 1:** Characteristics of included studies.

Study, country, design		Participants (N)		Participants details		Intervention		Control		Learning outcomes	
**Digital education versus usual learning**											
	Chaikoolvatana 2009, Thailand (RCT^a^) [30]		85		Pharmacy students		Interactive computer program for smoking cessation counseling		Classroom lectures		Knowledge and attitude toward intervention
	Gordon 2013, United States of America (RCT) [31]		215		Respiratory therapists, nurses, and nurse practitioners		Web-based smoking cessation education program		Usual traditional learning		Behavior, attitude, and self-rated efficacy in providing smoking cessation therapy
	Young 2002, Australia (RCT) [26]		53		Family physicians		Web-based distance learning module for delivering smoking cessation advice		Preventive care guidelines sent via postal mail		Knowledge, skill, readiness to change, and self-rated competence
	Galal 2015, United States of America (RCT) [32]		214		Pharmacy students		Use of student response systems (SRS) or “clickers” for instruction in a smoking cessation module		Instruction without student response systems		Learner’s attitude toward intervention
**Blended education versus usual learning**											
	Butler 2013, United Kingdom (cRCT^b^) [27]		53		General practitioners, and nurses		Web-based learning program with face-to-face trainings to deliver behavior change counseling in smoking cessation		Usual traditional learning		Patient reported changes in smoking behavior after health professionals training
	Hymowitz 2007, United States of America (cRCT) [29]		16		Pediatric residents		Hybrid CD-ROM/website training program and seminars series to deliver smoking cessation therapy		Usual learning with reading material on smoking cessation		Skill, practice, and behavior change
	Ockene 2015, United States of America (cRCT) [33]		1503		Medical students		Web-based multi-modal education and face-to-face trainings for smoking cessation counseling		Usual learning with traditional tobacco education in the medical curricula		Smoking cessation counseling skill and self-rated competence
**Digital education versus digital education**											
	Bonevski 1999, Australia (RCT) [34]		19		General practitioners		Computer-based program with performance specific feedback system for screening smoking behavior		Computer program without feedback system for smoking cessation behavior		Screening smoking behavior (smoking status classification)
	Stoner 2014, United States of America (RCT) [35]		92		Physicians, nurse practitioner, and physician assistants		Web-based multimedia training program for screening, brief intervention, and referral to treatment		Website with hyperlinks to downloadable reading materials		Knowledge, satisfaction, self-efficacy, and change in clinical practice
**Blended education versus digital education**											
	Brunette 2015, United States of America (RCT) [36]		46		Psychiatrists, advanced nurse practitioners		Videoconference educational outreach and use of printed slides, handouts, and questionnaires for cessation pharmacotherapy		Interactive in-person lecture with slides and handouts		Knowledge and attitude toward intervention
	Stolz 2012, Switzerland (RCT) [37]		129		Medical students		Self-directed Web-based module		Lectures with video demonstration on smoking cessation		Knowledge, skill, satisfaction, and self-rated smoking cessation counseling skill

^a^RCT: randomized controlled trial.

^b^cRCT: cluster randomized controlled trial.

Comparison interventions also varied across the studies. A total of 7 studies included a traditional learning control group: face-to-face workshops or lectures in 3 studies [33], reading materials in 1 study [29], and preventive care guidelines sent via postal mail in 1 study [26]. Furthermore, 2 studies did not specify details of the traditional learning intervention [27,31]. In addition, 4 studies evaluated others forms of digital education in the control group: computer-based offline learning in 3 studies [25,26,33] and Web-based learning in 1 study [35].

### Methodological Quality of Included Studies

**Figure 2:**
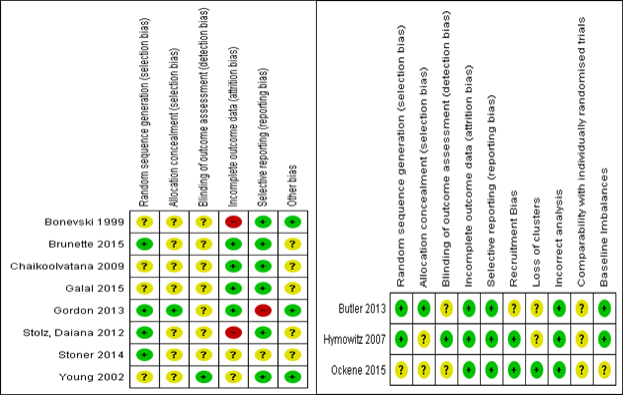
Risk of bias summary: reviewers' judgements about each risk of bias item for each included study (RCTs and cRCTs).

A total of 3 studies were judged as high risk of bias for at least 1 domain; all other studies were rated as unclear risk of bias. Concealment of treatment allocation and blinding of outcome assessors were particularly, poorly reported with only 2 studies judged as low risk of bias for each of these domains. Information on incomplete outcome data and selective outcome reporting was better reported with 2 studies judged as unclear risk of bias for incomplete outcome data and 1 for selective outcome reporting. Furthermore, 2 studies were judged as high risk of bias for incomplete outcome reporting and 1 for selective outcome reporting. No other domains were judged high risk of bias for any of the included trials (Figure 2). For cRCTs, none of the studies were judged as high risk of bias for any domain; all studies were judged as either low or unclear risk of bias for recruitment, loss of clusters, and incorrect analysis.

### Primary Outcomes

### Knowledge

**Figure 3:**
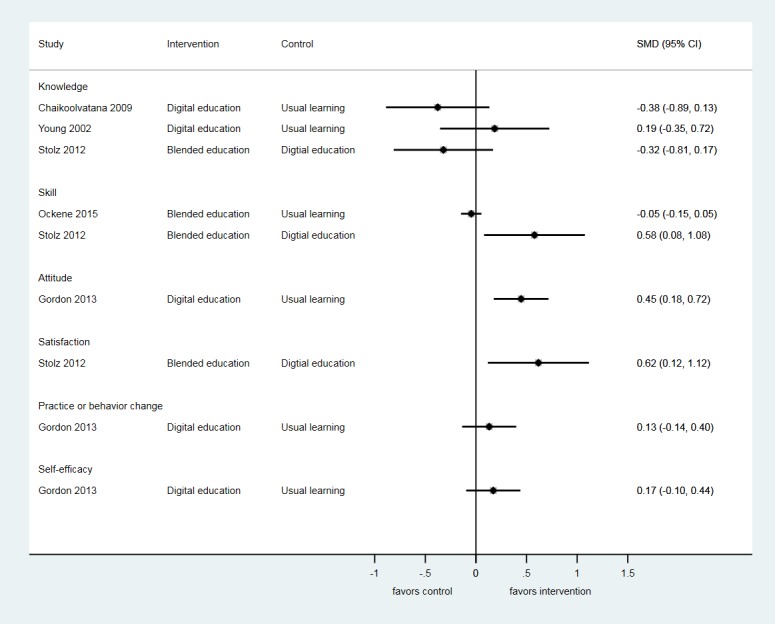
Forest-plot of outcomes showing standardized mean deviations (SMDs) and 95% CI for studies comparing digital education or blended education with usual learning.

A total of 5 studies (313 participants) assessed postintervention knowledge gain using multiple-choice questions (MCQs) [26,30,35,37] or questionnaires [36] (Multimedia Appendix 2). None of the studies used validated instruments to measure knowledge. Moreover, 3 studies provided quantitative data and found that knowledge gain was similar with digital education compared with usual learning and for blended education compared with digital education (Figure 3). Furthermore, 2 studies did not provide numerical data. One study compared 2 different types of digital education (Web-based multimedia training compared with a website with hyperlinks to downloadable reading material) and found no difference between the interventions [35]. The other study compared blended education with digital education and found no difference in postintervention knowledge between groups [36].

### Skill

A total of 5 studies (3293 participants) assessed postintervention skill to deliver smoking cessation therapy using objective structured clinical examination scores, [29,33,37] a Likert scale [26], or practitioner checklist [34] (Multimedia Appendix 2). Only 1 study used a validated assessment tool [33]. Furthermore, 1 study found that blended education was associated with greater improvement in skill for delivering smoking cessation advice compared with digital education alone (SMD=0.58, 95% CI 0.08-1.08) [37]. A second study found greater improvement in skill with blended education compared with usual learning (RR=2.04, 95% CI 1.51-2.76) [29]. However, a further study that compared blended education with usual learning found no difference between groups (SMD=−0.05, 95% CI −0.15 to 0.05) [33]. A study that compared 2 types of digital education involving a computer-based program with or without a performance specific feedback system found no difference in skill between groups (RR=1.01, 95% CI 0.96-1.08) [34].

Another study reported small improvements in postintervention skill of health professionals compared with usual learning; however, no numerical data were reported for quantitative analysis [26].

### Attitude

A total of 4 studies (532 participants) assessed postintervention attitude toward educational interventions [30,32] and new knowledge [31,36] using Likert scales [30-32] and a questionnaire [36] (Multimedia Appendix 2). None of the studies used a validated instrument.

Moreover, 1 study reported improved attitudes following digital education compared with usual learning (SMD=0.45; 95% CI 0.18-0.72) [31]. Furthermore, 3 studies did not provide quantitative data. In addition, 2 of these compared digital education to usual learning and reported positive attitudes toward digital education [30,32]. Another study reported no difference in postintervention attitude between blended education and digital education [36].

### Satisfaction

A total of 3 studies (415 participants) assessed postintervention satisfaction with the educational interventions using nonvalidated MCQs [37] and Likert scales [31,35] (Multimedia Appendix 2). Moreover, 1 study reported greater satisfaction after blended education compared with digital education (SMD=0.62, 95% CI 0.12-1.12) [37]. In addition, 2 studies did not provide any quantitative outcome data. Out of these, 1 reported higher postintervention satisfaction in digital education compared with usual learning [31]. Another study reported that postintervention satisfaction was higher with Web-based digital education compared with digital education through websites with hyperlinks [35].

### Secondary Outcomes

### Practice and Behavior Change

A total of 4 studies (650 participants) assessed postintervention practice and behavior change using nonvalidated surveys [29] and Likert scales [26,31,35,36] (Multimedia Appendix 2). Furthermore, 1 study reported higher postintervention readiness to change practice and behavior to help patients quit smoking with blended education compared with the usual learning (RR=1.58, 95% CI 1.25-2.00) [29]. Another study reported no difference in tobacco cessation–related behaviors between the digital education and usual learning groups (SMD=0.13, 95% CI −0.14 to 0.40) [31]. Furthermore, 1 study reported no difference in postintervention practice and behavior change between 2 modalities of digital education [35]. Similarly, no difference was observed between digital education compared with usual learning in another study [26,36]. No numerical data were reported for these 2 studies to be included in the quantitative analysis.

### Self-Efficacy

A total of 6 studies (1988 participants) assessed postintervention self-efficacy using nonvalidated questionnaires [30,37], Likert scales [26,31,35], and a 6 item checklist [33] (Multimedia Appendix 2). Moreover, 1 study reported no difference in postintervention self-rated smoking cessation counseling skill between blended education and digital education (RR=0.38, 95% CI 0.12-1.13) [37]. Another study reported no difference in self-efficacy scores toward providing tobacco cessation interventions in the digital education and usual learning group (SMD=0.17, 95% CI web 0.10 to 0.44) [31]. In 1 study, higher number of participants receiving blended education reported self-efficacy for performing smoking cessation-related counseling compared with usual learning (*P*<.05) [33]. In another study, change in self-efficacy between baseline and postintervention was significantly greater with digital education compared with usual learning (*P*=.03) [26]. Moreover, 1 study reported no difference in postintervention self-efficacy between 2 modalities of digital education [35]. In another study, where self-efficacy was measured only in the intervention group, computer-based program was reported to be effective in improving smoking cessation counseling skill by 73.34% of intervention group participants [30]. No numerical data were reported for these 4 studies to be included in the quantitative analysis [29,31,35,37].

### Patient Outcomes and Cost

There was 1 study which assessed postintervention patient outcomes such as smoking index, general health score, quality of life score, and the cost associated with the blended education intervention (Multimedia Appendix 2). The total cost including intervention and health care cost per practice was estimated to be US $2384. However, no quantitative data were reported [27].

## Discussion

### Overview

Our review included 11 studies that investigated the effectiveness of digital education for training health professionals to deliver smoking cessation therapy. No difference was found between digital education and traditional or usual learning. There was some suggestion that blended education results in greater improvements in satisfaction, skill, and knowledge compared with digital education alone. There was also some evidence for improved attitude following digital education compared with usual learning. Only 1 study reported patient outcomes and the setup cost of digital education. There were insufficient data to investigate what components of the digital education interventions were associated with the greatest improvements in learning outcomes. Studies were poorly reported, heterogeneous, assessed a broad range of different outcomes, and compared different types of interventions on the range of pre and postregistration health professionals. The findings of this review should therefore be interpreted with some caution.

### Strengths and Weaknesses

As far as we are aware, this is the first review to address the topic of digital education to train health professionals to deliver smoking cessation therapy. We followed best practice methods for systematic reviews, which attempted to minimize risk of bias and errors in the review process. We conducted a comprehensive sensitive search across a broad range of databases and included additional steps to identify unpublished studies such as searching trials registers, meeting abstracts and PhD theses, screened references of included studies, and contacted authors of abstracts for further information. It was not possible to formally assess the risk of publication bias because of the small number of heterogeneous studies included in our review, but given our extensive search, we consider it unlikely that relevant studies have been missed. Moreover, 2 independent reviewers were involved in all stages of the review process, standardized data extraction forms were used, and we used an accepted tool to assess the risk of bias in the included studies. This identified potential limitations in the included studies, particularly in reporting, which meant that many of the risk of bias domains were judged as unclear for the majority of studies. The included studies evaluated a broad range of interventions and outcomes; therefore, it was not appropriate to calculate summary effect estimates. The included digital education interventions mostly comprised asynchronous Web-based programs aimed at postregistration health professionals. For busy health professionals, digital education is a convenient avenue for fulfilling continuing medical education requirements and promoting knowledge and skill in particular areas in which they may not have previously had training in. Furthermore, 2 studies included an interactive or feedback component in the digital education intervention [34,36]. Evidence suggests that interventions with feedback and interactivity can enhance engagement and consequently the effectiveness of learning [38,39]. However, there were insufficient data available in our review to perform subgroup analyses or a more advanced statistical analysis to evaluate what components of the included interventions contributed to the greatest improvement in outcomes.

There were a number of limitations with the included studies. Reporting of the digital education interventions, especially the description of the intervention, aims and outcomes, pedagogical approach, and use of validated outcome assessment instruments was inconsistent across the studies. There was also a lack of baseline assessment in some studies, meaning that only postintervention data could be used in the analysis, potentially biasing results. Several studies did not report numerical data; therefore, they could not be formally included in our synthesis. We have included results available from these studies, but had additional data been available in the included studies, these would have allowed us to conduct a more informative analysis.

### Implications for Practice

When considering the implications of the findings of our review for practice, it is important to consider the implications of the differences in the effectiveness of different types of education. For forms of digital education that have additional benefits compared with standard education (eg, ability to target larger numbers of people, self-paced learning), showing that these are as effective as standard education is likely to be sufficient to recommend the use of these types of education. In contrast, for types of digital education where technology is supplementing standard face-to-face learning (eg, blended education or use of “clickers”), outcomes would need to be better with digital education than with standard education. For example, the study evaluating the use of clickers to give instructions during classroom lectures reported a positive impact of technology on learners’ attitudes, which consequently improved learning outcomes [32]. Our findings suggest that computer-based education, both Web-based and offline, is at least as effective as usual or traditional learning for smoking cessation therapy training in health professionals. This suggests that computer-based learning is an appropriate method to deliver training for health professionals. We found that blended education appears to offer additional benefits compared with digital education or traditional education alone. However, given the additional costs of this type of education, further studies on the cost effectiveness of blended education are needed before this can be recommended for use in practice. There were insufficient data from the studies included in our review to make specific recommendations regarding what types of digital education or components of digital education are likely to be most effective.

### Implications for Research

There is a need for further robust studies in a range of settings to determine the true potential of digital education to train health professionals to deliver smoking cessation therapy. More studies evaluating patient outcomes such as postintervention smoking cessation rates, smoking status, and abstinence are needed to assess the effectiveness of digital education for health professionals. Before recommending implementation of digital education programs, information on cost effectiveness, sustainability, as well as the direct and indirect costs such as time to develop as well as implement a smoking cessation module is needed. This will help policy makers to make practical recommendations and allocate resources appropriately. More research is needed to understand the feasibility of integrating digital smoking cessation training methods into the curriculum and continued medical education; research is also needed to understand the short-term and long-term effects in different geographical, socioeconomic, and cultural settings. In addition, sustainability, cost savings, and accreditation of digital smoking cessation therapies in health professional training need to be further researched.

Many digital education interventions in the included studies were based on smoking cessation guidelines. However, validation of the course content and use of underpinning learning theories to develop the pedagogy were lacking in most of the studies. Largely, the studies focused on integrating the new technology into the existing curriculum as opposed to using learning theories to design the digital education intervention for successful delivery of education. An increasing body of evidence reveals that theory-based interventions have greater impact than those that are not based on theory. Appropriate use of learning theory and pedagogy framework along with sound methodology can enable more robust studies to be conducted on digital education with research questions adequately addressed through theoretically informed research design, data collection, and analysis [40-42]. Future studies should therefore focus on developing, delivering, and evaluating digital education with a strong pedagogical foundation.

Digital education can contribute significantly to the World Health Organization (WHO) mission to transform and scale up health professionals’ education by filling the medical education divide between low- and high-income countries [43]. Given the greater prevalence of smoking in low-income countries and lesser awareness of harms of smoking compared with high-income countries, being able to train health professionals to deliver smoking cessation advice is of particular importance for low-income countries. However, none of the studies included in our review assessed the use of digital education in such resource-constraint settings. This is an important area for future research.

We did not identify any studies on advanced educational technologies for digital education such as mobile learning, virtual patients, virtual reality environments, or serious gaming, which have the potential to transform education for health professionals. The lack of smoking cessation studies evaluating these educational technologies makes it difficult to make recommendations for integrating such pivotal digital technologies into health professional education. In the future, it is important to have a more detailed reporting of different components of digital education interventions to allow for a more thorough analysis of the most active and effective components.

### Conclusions

Digital education appears to be at least as effective as usual or traditional learning in improving health professionals’ knowledge and skill for delivering smoking cessation therapy. This suggests that digital education is an appropriate method to deliver training for health professionals on how to deliver smoking cessation therapy. However, limitations in the evidence base mean that these conclusions should be interpreted with some caution. There was insufficient evidence to determine what components of digital education are associated with the greatest improvements in outcomes, although there was some evidence that blended education may be more effective than either digital education or usual learning alone.

## Appendix

Multimedia Appendix 1 MEDLINE (Ovid) search strategy.

Multimedia Appendix 2 Results on included studies.

## Abbreviations

cRCT: cluster randomized trial
MCQ: multiple-choice question
RR: relative risk
SMD: standardized mean difference
WHO: World Health Organization
