# Supplementary material for: Digital Education for Health Professions on Smoking Cessation Management: Systematic Review by the Digital Health Education Collaboration
Source: J Med Internet Res. 2019 Mar 4;21(3):e13000. doi: 10.2196/13000 (PMC6421523; doi:10.2196/13000)
Supplement: Multimedia Appendix 2 [file jmir_v21i3e13000_app2.pdf]

Multimedia Appendix 2: Results of Included Studies

Studies	Outcomes						
	Knowledge	Skill	Attitude	Satisfaction	Practice and Behavior change	Self-efficacy	Cost
**Digital education versus usual learning**							
Chaikoolvatana 2009 [36]	Knowledge of 5As[a] and case scenarios of smoking cessation counselling **Post-test MCQ**	-	-	-	-	Self-rated smoking cessation skill **Questionnaire** *Participants (73.34%) reported improvements in counselling skill*	-
Galal 2015 [37]	-	-	Attitude towards using teaching tool (SRS) **Likert scale**	-		-	-
Gordon 2013 [32]	-	-	Attitude towards training **Likert scale**	Satisfaction survey to assess website design, organization, and ease of use **Likert scale**	Change in behavior (sum of 4 factors - ask, assess, advice, and assist) **Likert scale**	Self-rated efficacy towards providing tobacco cessation interventions **Likert scale**	-

				Higher satisfaction with the training			
Young 2002 [29]	Knowledge of health effects of smoking **Post-test MCQ**	Smoking cessation techniques **Likert scale** *small improvements in intervention group*	-	-	Readiness to change (interest in changing current practice) **Likert scale** *no difference between two groups*	Self-assessed competence in smoking cessation advice **Visual antilog scale** *Significantly higher self-efficacy in intervention group (p=.03)*	-
**Blended education versus usual learning**							
Butler 2013 [31]	-	-	-	-	-	-	**Cost per practice** £ 1597.40
Hymowitz 2007 [28]	-	Interviewing and tobacco intervention skill **OSCE scores**	-	-	Readiness to change (feeling prepared to help patients quit smoking) **Survey**	-	-
Ockene 2015 [34]	-	Tobacco treatment skill	-	-	-	Self-reported skill for behavior and	-

		**OSCE scores**				pharmacotherapy counselling **Questionnaire** *Higher self-efficacy in intervention group* (p<.05)	
**Digital education versus digital education**							
Bonewski 1999 [27]	-	Smoking status classification **checklist**	-	-	-	-	-
Stoner 2014 [33]	Knowledge of SBIRT core curriculum and motivational counselling **Post-test MCQs** *knowledge improvement with training*	-	-	Post-training satisfaction **Questionnaire** *Higher satisfaction with the training*	Clinical practice behavior change **Likert scale** *no difference between two groups*	Self-efficacy for counselling patients **Likert scale** *no difference between two groups*	-
**Blended education versus digital education**							
Brunette 2015 [30]	Knowledge about treating nicotine dependence **Post-test Questionnair-e** *no difference between two groups*	-	Attitude towards video-conference sessions **Post-test Questionn-aire** *no difference*	-	-	-	-

			between two groups				
Stolz 2012 [35]	Knowledge of health effects of smoking **Post-test MCQ**	Smoking cessation counselling skill **OSCE scores**	-	Post-training satisfaction **Post-test MCQ**		Self-rated smoking cessation counselling skill **Questionnaire**	-

Footnotes: [a] = ask, advise, assess, assist and arrange; OSCE = objective structured clinical examination; SMD = standardized mean difference; MCQ = multiple choice question; RR = Relative Risk
